# Auxin treatments protect male reproductive development against cold stress

**DOI:** 10.1093/plphys/kiae162

**Published:** 2024-03-19

**Authors:** Joke De Jaeger-Braet

**Affiliations:** Assistant Features Editor, Plant Physiology, American Society of Plant Biologists; Department of Developmental Biology, Institute of Plant Science and Microbiology, University of Hamburg, Hamburg 22609, Germany

With the ongoing global climate change, plants are more often exposed to temperature extremes, both heat and cold stress. Plant reproduction, which is crucial for genetic diversity and seed production, is one of the developmental processes most sensitive to changing environmental conditions.

Inside the male reproductive organ, which consists of a pollen sac (also called anther) and a supporting filament, mature pollen is generated. During microsporogenesis, pollen mother cells undergo meiosis to produce 4 haploid microspores. With the onset of microgametogenesis, those uninucleate microspores undergo 2 rounds of mitosis. The first mitotic division results in the development of binucleate pollen containing 1 generative and 1 vegetative cell. The second mitosis of the generative cell leads to mature pollen with 2 sperm cells and 1 vegetative cell (Fig.). The quantity and quality of a seed set is highly dependent on successful pollen development, and environmental stresses can cause drastic reductions in pollen fertility.

**Figure.**
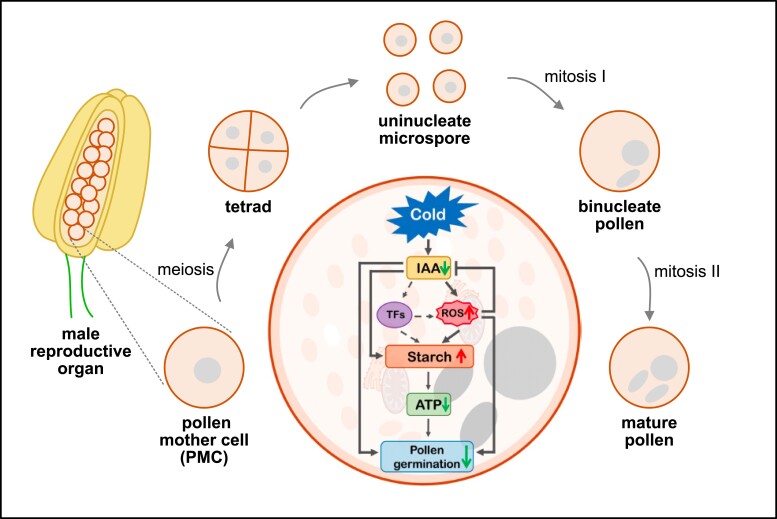
A schematic overview of male reproductive development and a proposed model for cold stress response during pollen development. A male reproductive organ consists of an anther (yellow) with a filament (green). Inside the anther, reproductive cells can be found (orange). The regulatory pathway response to cold stress during pollen development as proposed by (Liu et al. 2024) is surrounded by a schematic representation of male reproductive development.

Both microsporogenesis and microgametogenesis are known to be sensitive to cold stresses. Exposure of meiotic cells to low temperatures affects cytokinesis with the formation of aberrant phragmoplast like structures in Arabidopsis (*Arabidopsis thaliana)* and wheat (*Triticum aestivum*) (Tang et al. 2011; De Storme et al. 2012). The end of meiosis, that is, the tetrad stage, or the transition to early uninucleate microspores is found to be most cold stress sensitive in rice (*Oryza sativa*) and soybean (*Glycine max*), subsequently affecting pollen development (Oliver et al. 2005; Ohnishi et al. 2010). It has become clear that low temperatures affect male reproductive development but the underlying molecular mechanisms are not yet fully understood.

During pollen development, the reproductive cells are embedded in the anther, surrounded by a cell layer, called the tapetum, which is an important source of callose synthase, sucrose, and other nutrients for the reproductive cells. Sugar is transported from the tapetum to developing pollen to synthesize starch. Starch accumulation is initiated after the first mitosis, reaching a maximum in binucleate pollen. Starch then gets degraded into soluble sugar, which in turn is proposed to be an energy source required for pollen germination (Chen et al. 2023).

In this issue of Plant Physiology, Liu et al showed that in Chinese cabbage (*Brassica campestris*), cold stress decreases callose deposition during the tetrad stage. The temporal callose synthesis and degradation are required for tetrad formation and mature pollen release. Further, cold stress during binucleate pollen delayed degradation of the tapetum, which leads to starch overaccumulation in mature pollen and altered pollen exine layer formation, the protective cover of pollen. Low temperatures disturb the energy supply and metabolism, causing reduced pollen fertility. Germination assays could further reveal reduced germination rates of pollen cold stressed during pollen development, which subsequently leads to decreased seed set (Liu et al. 2024).

To elucidate the molecular pathways affected by cold stress, Liu et al. performed transcriptome and metabolome analysis of cold stressed anthers. The differentially expressed genes were mainly involved in energy metabolism and matched cytological observations. For example, the expression of starch-related genes was altered upon exposure to low temperatures, and an enhanced starch accumulation was found in mature pollen that originates from cold-treated cells during tetrad stage. These results led to the hypothesis that cold stress causes inefficient decomposition of starch. The starch decomposition is required for energy production and sugar used for callose synthesis (Fig.) (Liu et al. 2024).

The plant hormone auxin is required during pollen development, and recently it was shown in barley (*Hordeum vulgare*) that auxin synthesis in pollen controls the accumulation of starch by enhancing carbon metabolism to generate ATP (Cheng et al. 2006; Amanda et al. 2022). The wave of auxin synthesis is required to modify the energy metabolism and control starch accumulation during pollen maturation (Amanda et al. 2022; Mudgett and Zhao 2022). Liu and colleagues further showed that exposure to cold stress reduces the expression of auxin biosynthesis genes and auxin levels in microspores, which coincides with altered starch accumulation, leading to reduced pollen germination (Liu et al 2024).

Even more interestingly, auxin treatment at low concentrations before cold stress could increase callose deposition at the tetrad stage and effectively improves pollen fertility. These results suggest that exogenous auxin can improve pollen development tolerance to cold stress (Liu et al. 2024).

Revealing the molecular effect of cold stress during reproductive development leads to a better understanding of stress resilience in plants. The work of Liu and colleagues brought novel mechanistic insights to cold response during pollen development, which is of great potential for breeding programs focusing on environmental stress tolerance in crops to ensure seed yield and quality (Fig.).

## Data Availability

No new data were generated or analysed in support of this NAV.

## Dive Curated Terms

The following phenotypic, genotypic, and functional terms are of significance to the work described in this paper:

starch CHEBI: CHEBI:28017

ATP CHEBI: CHEBI:15422
